# Nanoimprinted High-Refractive Index Active Photonic Nanostructures Based on Quantum Dots for Visible Light

**DOI:** 10.1038/s41598-017-17732-0

**Published:** 2017-12-15

**Authors:** Carlos Pina-Hernandez, Alexander Koshelev, Scott Dhuey, Simone Sassolini, Michela Sainato, Stefano Cabrini, Keiko Munechika

**Affiliations:** 1 aBeam Technologies, 22290 Foothill Blvd, St. 2, Hayward, CA 94541 USA; 2 The Molecular Foundry, Lawrence Berkeley National Laboratory, 1 Cyclotron Road, Berkeley, CA 94720 USA

## Abstract

A novel method to realizing printed active photonic devices was developed using nanoimprint lithography (NIL), combining a printable high-refractive index material and colloidal CdSe/CdS quantum dots (QDs) for applications in the visible region. Active media QDs were applied in two different ways: embedded inside a printable high-refractive index matrix to form an active printable hybrid nanocomposite, and used as a uniform coating on top of printed photonic devices. As a proof-of-demonstration for printed active photonic devices, two-dimensional (2-D) photonic crystals as well as 1D and 2D photonic nanocavities were successfully fabricated following a simple reverse-nanoimprint process. We observed enhanced photoluminescence from the 2D photonic crystal and the 1D nanocavities. Outstandingly, the process presented in this study is fully compatible with large-scale manufacturing where the patterning areas are only limited by the size of the corresponding mold. This work shows that the integration of active media and functional materials is a promising approach to the realization of integrated photonics for visible light using high throughput technologies. We believe that this work represents a powerful and cost-effective route for the development of numerous nanophotonic structures and devices that will lead to the emergence of new applications.

## Introduction

The development of active photonic devices with quantum dots (QDs) as active media in the visible wavelength range promises a revolution in a wide variety of areas from biochemical sensing to quantum computing. Novel active photonic devices including nanolasers^1–4^ and light emitting diodes^5–9^ have been demonstrated over the last years, but are primarily limited to research laboratories owing to the expensive and complex fabrication of nanoscale features with high fidelity and precision. In particular, active photonic devices which operate in the visible region impose additional challenges as the dimensions of the features are approximately λ/4n (λ, wavelength, n, the refractive index). At these length scales, standard nanofabrication methods such as photolithography, e-beam lithography, and molecular beam epitaxy are either prohibitively expensive, very slow or not compatible with optically active materials. Some processes require dry etching steps with plasma exposure or operating at elevated temperature, which can easily be detrimental to preserving the optical properties of active materials. Solving these issues will be the key to developing a new path towards a high throughput and large scale manufacturing of active nanophotonic devices. Here we report a dramatically simplified method to fabricate active photonic devices in the visible region by direct nanoimprinting of high-refractive index materials integrated with colloidal quantum dots, demonstrated on active one (1-D) and two (2-D) dimensional photonic crystals.

The direct patterning of functional materials, while preserving the properties of the active materials, represents a robust and practical solution to fabricate active photonic devices. It is a powerful strategy as it enables to fabricate photonic devices in a single step processing with the ability to tailor specific properties (e.g. emission wavelength) of a device starting at the molecular level. Following this general scheme, varieties of nanostructured devices have been fabricated by patterning polymeric materials with embedded quantum dots using different nanofabrication technologies including nanoimprint lithography^10–13^, transfer replica^14^ and inject printing^15,16^. However, the development of novel optical elements by direct patterning has been restricted mostly to IR outside of the visible region, mainly due to limited availability in the high-refractive index polymeric materials above n = 1.50. High refractive index of the patterned material is essential for the future applications. The main advantage of a high refractive index material is the ability to realize compact photonic lightwave circuits. High refractive index is also essential to achieve high Q factor in 2D cavities^17^, since a complete photonic bandgap is needed to keep the light inside the cavity. In addition to that, high refractive index reduces the cavity length for nanocavity light sources and allows the fabrication of photonic elements such as ring resonators that rely on tight waveguides turns.

Nanoimprint lithography (NIL), a high throughput approach with molecular resolution and suitable for large-scale nanofabrication^18,19^, has demonstrated capabilities for the patterning of functional materials with high refractive index (n > 1.9)^20–25^. Nanoimprint of high refractive index materials with integrated quantum dots can lead to the realization of novel active photonic devices that can trigger the full potential in this field. Several high refractive index materials have been nanoimprinted mainly based on TiO_2_ sol-gels^20–25^ exhibiting promising attributes in their optical properties such as high index value with high optical transparency. Unfortunately, most of these high index materials, regardless of their specific chemical compositions, require very harsh processing conditions, i.e., high temperatures (~400 °C) or exposure to extremely high UV light intensities to achieve the desired optical properties which are detrimental for preserving the optical properties of the active materials.

**Figure 1:**
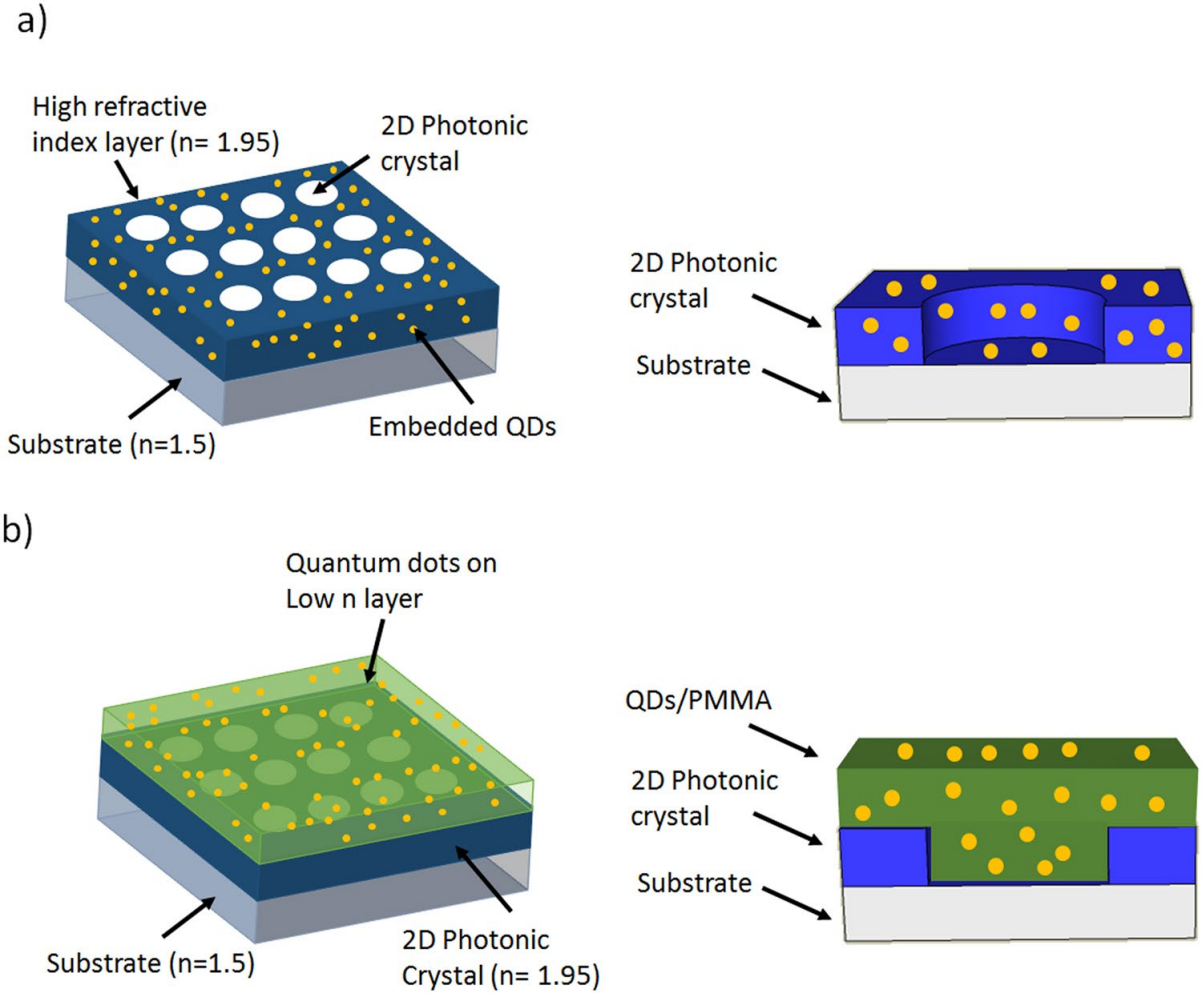
Schematics of: (**a**) A printed photonic crystal with embedded CdSe/CdS QDs, (right) cross-section of a single photonic crystal. (**b**) A photonic crystal with a slab of QDs/PMMA on top, (right) cross-section of a single photonic crystal.

In this report, we present novel nanoimprinting of high-refractive index materials integrated with quantum dots nanocrystals to fabricate active photonic devices with high resolution and fidelity. As a model system, one (1-D) and two (2-D) dimensional photonic crystals and nanocavities were nanoimprinted following a simple process and demonstrated enhanced photoluminescence and density of optical states (DOS) modification. The printed devices showed excellent optical properties and the processes are suitable for large size scalability. The fabrication of the photonic nanostructures was performed following two different techniques: 1) embedding thermally stable CdSe/CdS core/shell quantum dot nanocrystals^26–28^ in a high refractive index (n = 1.95) matrix (Fig. 1a) to fabricate photonic crystals with enhanced photoluminescence; 2) patterning a high refractive index material to create photonic nanocavities with the subsequent coating of a QD/polymer composite film (Fig. 1b) to generate active devices that produce photoluminescence with designed spectral properties through the change in DOS.

The photonic crystals for enhanced photoluminescence were fabricated by patterning a hybrid nanocomposite consisting of a high n material with embedded quantum dots, which act as the active component. The general procedure involves a reverse imprint method we previously developed to faithfully replicate high refractive index nanostructures with an accurate control of dimensional shrinkage^29^. The original process required an annealing step at 400 °C which led to large shrinkage (80% in the vertical direction) of the high n material and required a 3-layer step coating to preserve the pattern transfer fidelity.

The high refractive index material was then modified to achieve two outstanding properties: 1) less shrinkage, 30% in the vertical direction, which reduces the number of required coating cycles from three to only one; 2) a lower anneal temperature, 180 °C, instead of 400 °C, which is compatible with thermally stable quantum dots nanocrystals. These properties were achieved by tailoring the content of the organic and inorganic precursors composing the high refractive index material^21^. Based on this approach, a single layer of a functional hybrid high refractive index material is coated on top of a nanostructured mold by a spin coating process followed by an annealing step at 180 °C for 10 min to achieve the refractive index of 1.95 at a wavelength of 590 nm. This significant reduction in the annealing temperature preserves the quantum dots properties which is essential for active applications (Supplemental Information 1). The coated mold is brought in contact with a substrate to transfer the replicated nanostructures using a UV curing polymer as an adhesive. The hybrid nanocomposite contained 5% of quantum dot by weight and can be coated with good uniformity to form crack free films through a spin coating process. Composite formulations with higher contents of quantum dots can be prepared, but the films present high surface roughness, not suitable for device fabrication.

**Figure 2:**
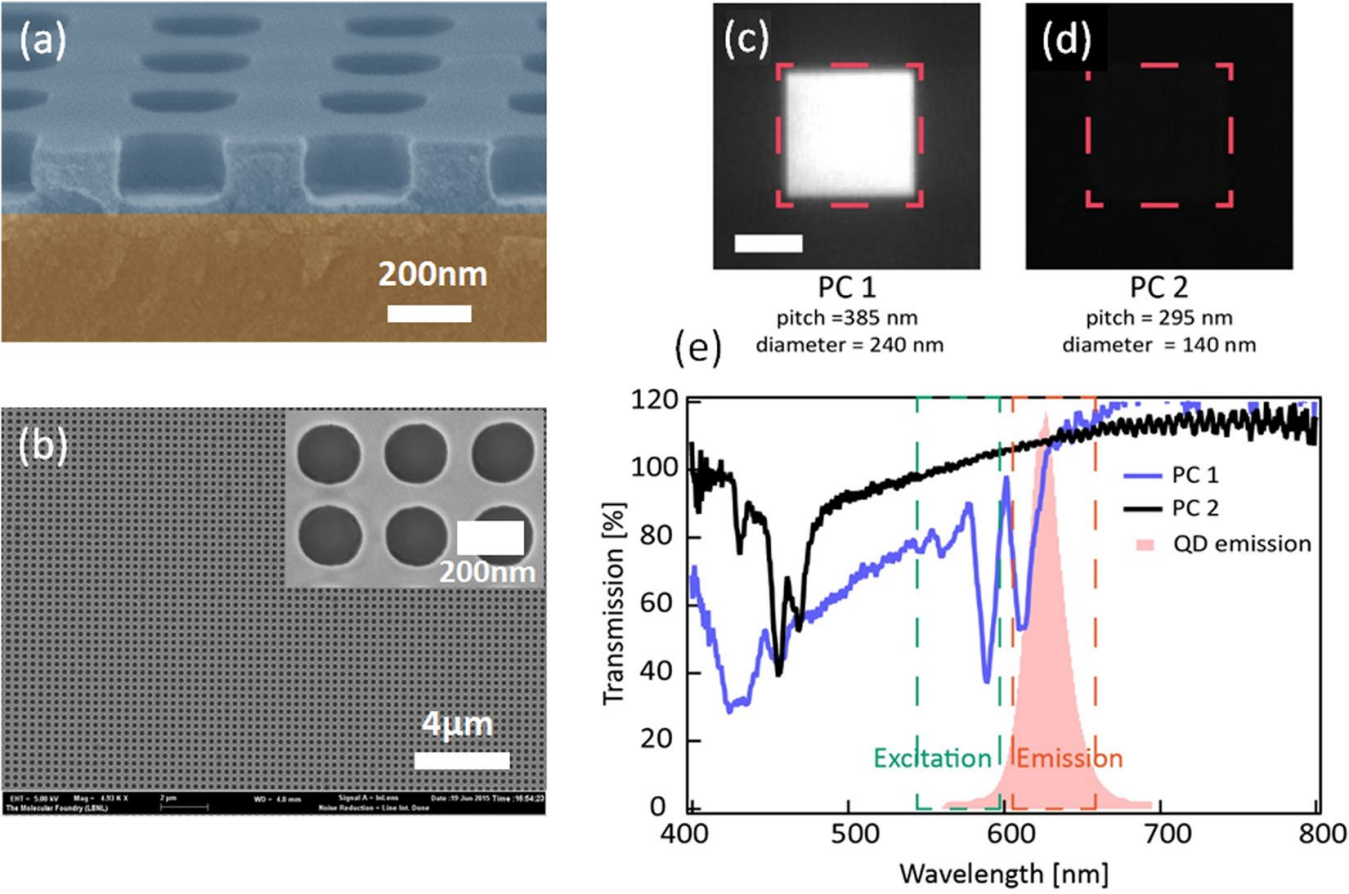
(**a**) SEM cross-section image and (**b**) top view image of an imprinted photonic device with embedded CdSe/CdS QDs. Fluorescence micrographs taken from different printed photonic crystals with QDs: (**c**) PC 1 (pitch = 385 nm, diameter = 240 nm) and d) PC2 (pitch = 295 nm, diameter 140 nm). Red dashed lines highlight the location of the printed photonic crystals. (**e**) Transmission spectra of PC1 and PC2. Green and Red dashed lines indicate the wavelengths used to excitation (green) and collection (red) of QD photoluminescence. Red shaded spectrum is the photoluminescence of the QDs used in this study. (Scale bar in Fig. 2c = 50 µm).

In order to explore the validity of the printed 2D photonic crystal devices, transmission measurements and fluorescence microscopy were performed. The absorption efficiency of the excitation light produced by the quantum dots in non-patterned films is very low due to its small thickness (film thickness <100 nm) which produces emitted fluorescence with low brightness. Increasing the QDs absorption efficiency would benefit many applications, such as display technology^30^, light emitting devices^31^ and solar cells^32^. One approach to enhancing absorption is to create a Guided Mode Resonance (GMR) effect to increase the length of interaction between the excitation light and a patterned layer^33^. The GMR effect occurs when periodic structures scatter incident light wave in phase relative to the waveguide mode. The incident light is coupled into a waveguide. Light coupling efficiency significantly increases the propagation lengths inside the waveguide material which gives rise to the absorption of light by QDs and with a subsequent fluorescence process. The device was structured with a 2D photonic crystal with a high refractive index (n = 1.95) patterned on top of a low refractive index (n = 1.5) glass substrate. Photonic crystals with a variety of diameters and pitches were patterned in a simple step by reverse imprint lithography. Nanostructures with a diameter of 200 nm were easily obtained as can be observed in the photonic crystals presented in Fig. 2a and b where patterns with high fidelity and sharp definition are shown. Fluorescence intensities of QDs were compared between the regions with and without printed photonic crystals, and a fluorescence enhancement factor of ~10 was measured from the patterned regions. The fluorescence enhancement was calculated taking into account the light intensity inside the photonic crystals and based corrected with respect to the non-patterned areas. The volume of quantum dots inside and outside the printed regions was quantified and considered in the calculation. Furthermore, it was found the degree of photoluminescence enhancement is highly frequency dependent. The largest fluorescence enhancement is observed in the photonic crystals with the GMR that spectrally overlaps with the excitation frequency and emission frequency of the QDs used for this study. In contrast, when there is no spectral overlap, no fluorescence enhancement is achieved (Fig. 2c–e). These results show for the first time photoluminescence enhancement in nanoimprinted photonic crystal with quantum dots embedded in a high refractive index matrix.

**Figure 3:**
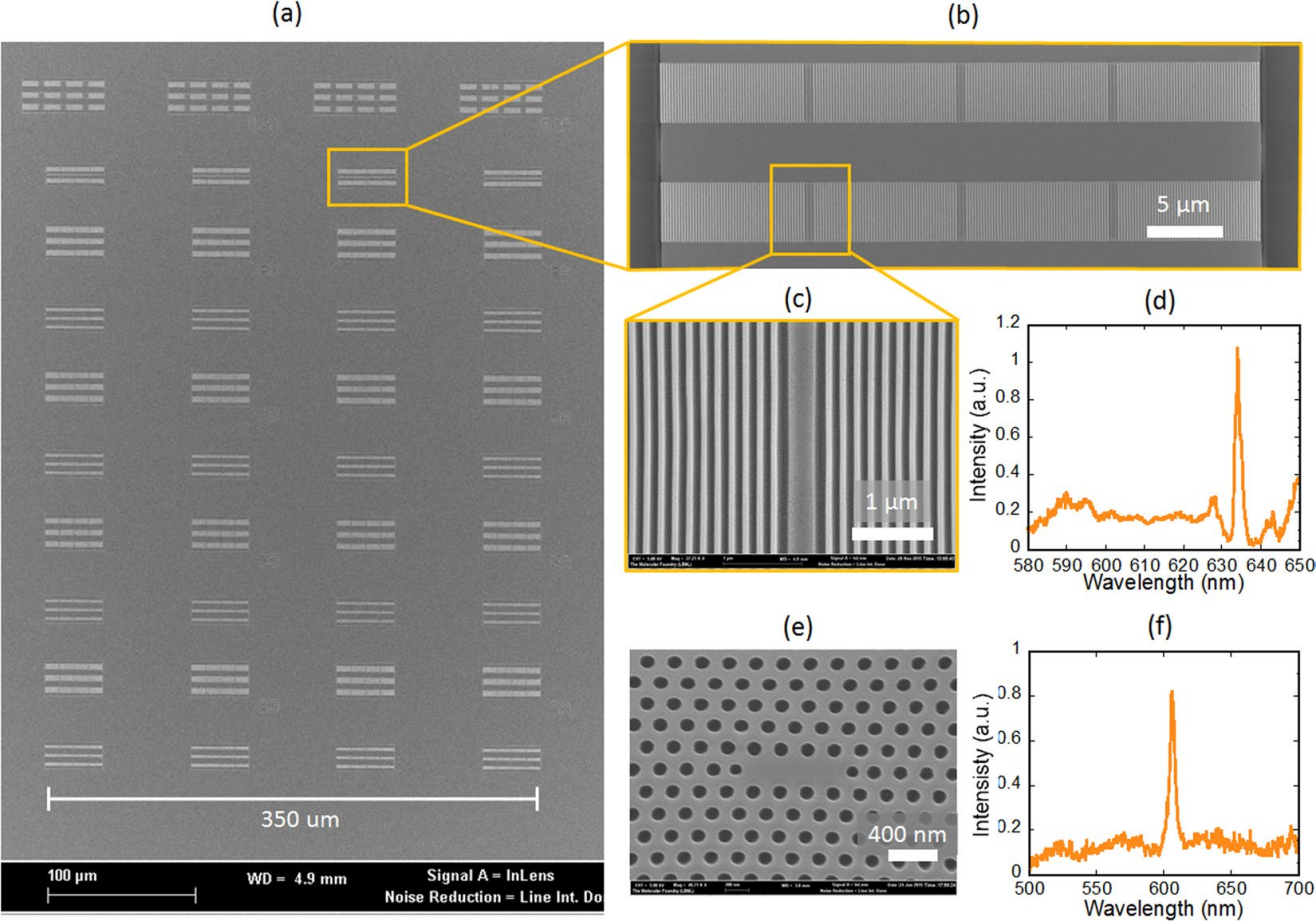
(**a**) SEM image of an array of nanoimprinted cavities with a variation of pitch and trench size. (**b**) Low magnification SEM image of a 1D nanocavity array printed in a high refractive index material; (**c**) high magnification SEM image 1D nanocavities and (**d**) transmission intensity in cross-polarization of the 1D cavity with a quality factor of 1000. (**e**) High magnification SEM image of a 2D nanocavity and (**f**) transmission intensity in cross-polarization of the 2D nanocavity.

The high refractive index of the functional material creates an opportunity to exploit the different type of structures such as photonic crystal cavities for localization of the light. A photonic crystal cavity is a defect inside a photonic crystal structure with a bandgap. Compared to the guided mode resonance structures, their fabrication is extremely challenging as the typical length scale for the photonic crystal is approximately two times smaller (λ/4 instead of λ/2). With our nanofabrication process, we were able to successfully imprint arrays of photonic crystal nanocavities with sub-100nm resolution (Fig. 3) over a large area with high quality. An array of nanocavities with varying pitch and trench is presented in Fig. 3a demonstrating that the array is imprinted with no defects. Higher magnification SEM images of the 1-D nanocavities are presented in Fig. 3b and c. Figure 3d shows a representative 1-D cavity resonance spectrum measured in cross-polarization with a quality factor of 1000. An imprinted 2-D nanocavity along with its corresponding transmission intensity in cross polarization are presented in Fig. 3e and f. The measured Q-factor of the cavity is 135, which is close to the simulated Q factor of 155. The low Q factor is the result of an insufficient refractive index contrast, even with the use of high refractive index material. These results show the capabilities of the proposed process and functional material to fabricate complex heterogeneous geometries with high resolution and fidelity. The high refractive index composite we developed is a hybrid organic-inorganic material consisting of a TiO_2_ phase distributed in an amorphous polymeric phase. Although crystalline TiO_2_ presents lower transmission at the UV region due to its bandgap, the wavelength of interest for this report lays above 500 nm. The crosspolarization transmission measurements demonstrated high Q factor of the cavity (at least 1000), which indicates high transparency of the material at the wavelengths of interest.The photonic nanocavities with a refractive index of 1.95 were fabricated following one of the two previously described methods: 1) print the functional hybrid nanocomposite with embedded quantum dots and 2) print a high-refractive index nanocavity and coat it with a layer of QDs nanocrystals embedded in PMMA. Both of these methods were appropriate for the fabrication of 1-D and 2-D photonic crystal nanocavities having suitable quality factors. To the best of our knowledge, it is the first demonstration of optical nanocavities nanoimprinted in a high refractive index material.

**Figure 4:**
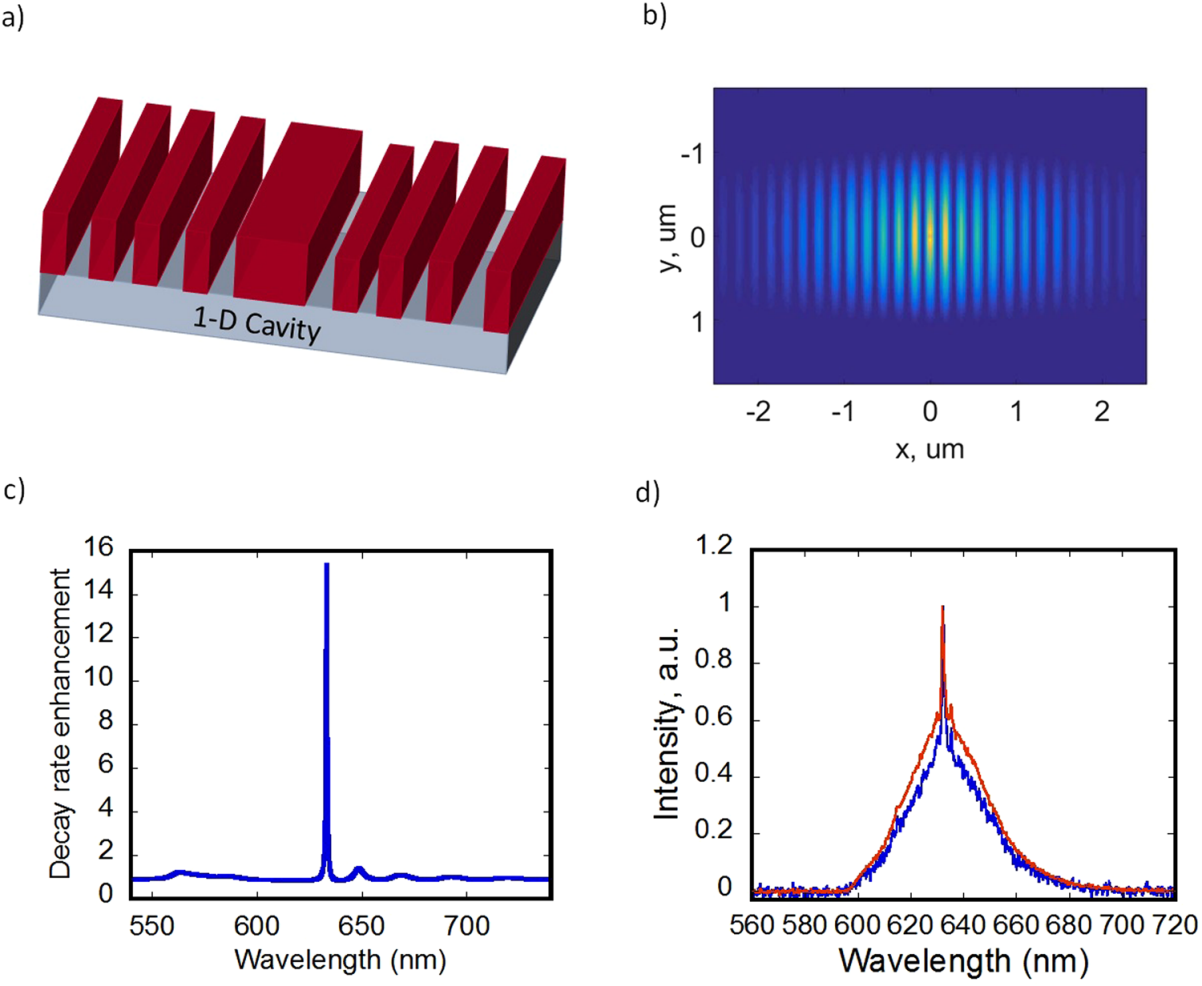
(**a**) Schematics of a 1D photonic crystal nanocavity, (**b**) field distribution of the resonant mode of 1D nanocavity obtained using commercial 3D FDTD code, (**c**) simulated ratio of the PL decay rate in the cavity to the decay of quantum dots outside the cavity, and (**d**) experimental photoluminescence spectrum of the nanocavity.

To simulate the resonant effects inside the fabricated nanocavities we used a commercial 3D FDTD code^34^. A 1D photonic crystal cavity was chosen (Fig. 4a) due to their high Q-factors which are possible to obtain even with a relatively low effective refractive index contrast between etched and non-etched regions^35^. The cavity is created inside a photonic crystal (pitch 190 nm, trench width 80 nm, height 160 nm) by removing the central trench. The simulated mode field profile of the transverse electric (TE) cavity mode is shown in the Fig. 4b. The photonic crystal cavity creates an increased local density of optical states for the resonant wavelength. The increase in DOS leads to the change in the lifetime of the photoluminescence which, in turn, leads to the PL spectrum modification. The simulated fluorescent decay rate is shown in Fig. 4c. The decay rate has a narrow peak that corresponds to the cavity resonance wavelength. Experimental verification of the decay rate modification is presented in Fig. 4d. The Fig. 4d shows measurements of PL spectrum taken from the center of the cavity. The measured spectrum shows a narrow peak at a resonant wavelength on top of a typical QDs spectrum. The observed PL narrowing and intensity enhancement suggest that our structures are able to modify the optical density of states within the cavity region^36–38^. The FWHM of the resonant peak is ~1 nm, which correspond to the Q factor of ~600.

**Figure 5:**
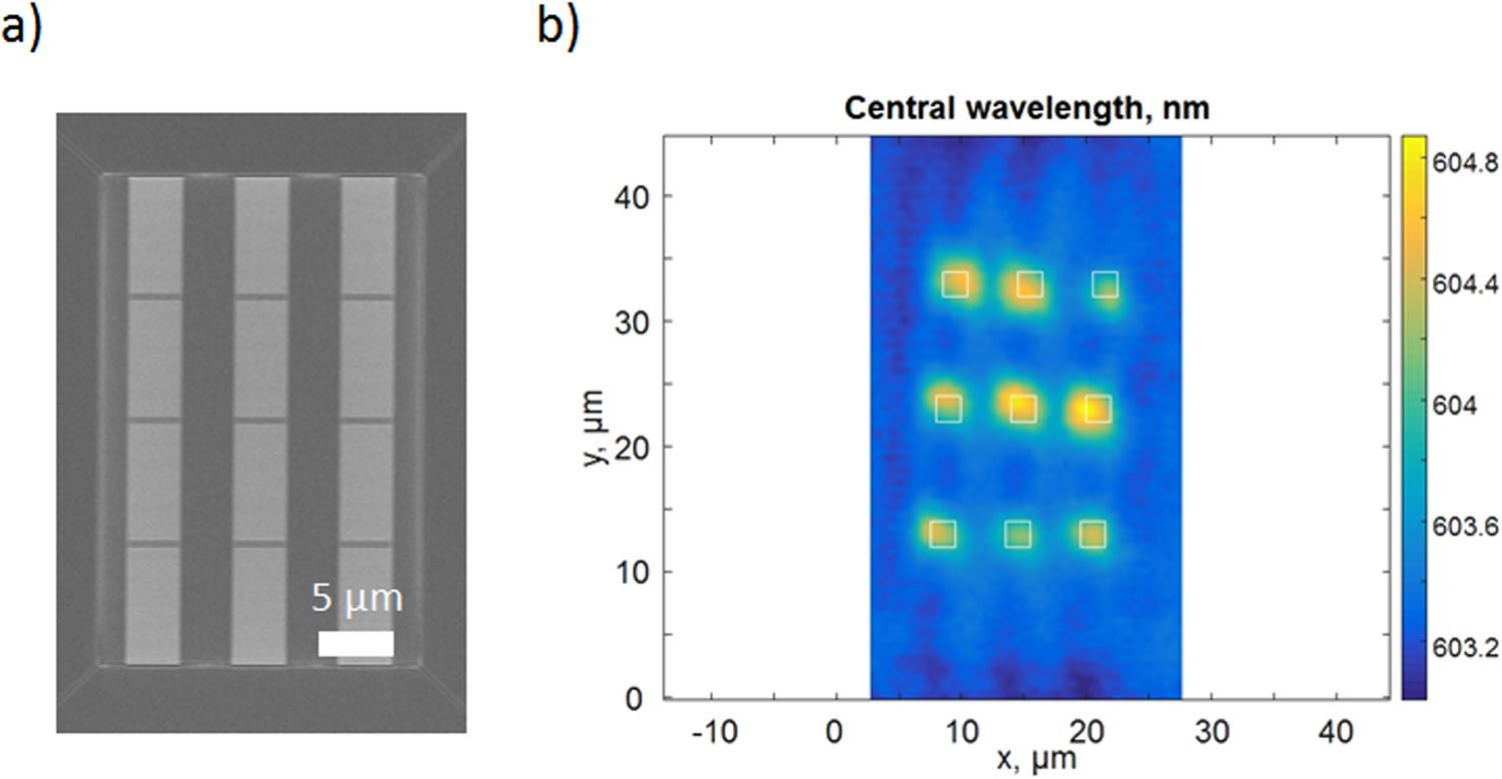
(**a**) Array of 1D photonic crystal nanocavities and (**b**) PL central wavelength distribution obtained in confocal scan measurements of the 1D nanocavity. FDTD simulation of the PL decay rate, that leads to the spectrum shift is shown in the Fig. 4c.

The photoluminescence spectrum of the imprinted nanocavities with integrated QDs was measured using a confocal microscope setup as described in detail in the method section. The excitation was performed using 470 nm picosecond laser pulses. The 1-D Photonic nanocavities were patterned in a high refractive index material (n = 1.95) and coated with a thick layer (2 µm) of a quantum dots/PMMA composite (Fig. 5a). The typical results of the confocal scan measurements are shown in Fig. 5b. As can be seen from the results, the presence of the cavity modifies the photoluminescence spectrum as observed by a shift of the central wavelength in the Fig. 5b distribution. The spectrum shift is only observed in the cavity region and consistent across all the array of printed nanocavities. The size of the area with the modified spectrum is ~3 µm, which is consistent with the cavity mode area (Fig. 4b).

This paper demonstrates that the nanoimprinting of high refractive index materials with quantum dots offers extraordinary capabilities for realizing active nanophotonic devices for visible wavelength. High-temperature resistant CdSe/CdS quantum dots and the novel high-refractive index material which require mild processing conditions were combined to form a novel hybrid nanocomposite and used to nanoimprint various photonic devices with sub-100 nm resolution and high fidelity. To demonstrate an active device, we have successfully patterned 2D photonic crystals and nanocavities which demonstrated enhanced photoluminescence due to increased QD absorption associated with GMR, and PL spectrum modification due to a change in the local density of states inside the nanocavity. It is also the first time photonic nanocavities with high Q factors are imprinted in a high refractive index material. This work represents a powerful and cost-effective route for the development of numerous nanophotonic structures and devices that will lead to the emergence of new applications. Advanced applications are under current development including optically pumped nanolasers and high-efficiency light emitting diodes.

## Methods

### Master mold fabrication

Master molds consist of hydrogen silsesquioxane (HSQ) nanostructures were directly patterned by electron beam lithography on silicon substrates. HSQ resist (Dow Corning®) were spin coated with a thickness between 80 and 160 nm.

### Photonic crystals and nanocavity fabrication with incorporated QDs

A solution of quantum dots nanocrystals mixed with a hybrid organic-inorganic TiO_2_ based material (aBeam Technologies’ proprietary material^39^) was used for the process. The content of QDs in the mixture was 4% by weight. The solution was spin coated on top of an HSQ master mold to create a thin film. The coated master mold was heated on a hot plate at 180 °C for 10 min and then reverse imprinted on a glass substrate with the help of an adhesive polymer to produce the nanoimprinted photonic structures.

### Nanocavity fabrication with integrated QDs

A hybrid organic-inorganic TiO_2_ solution was spin coated on top of an HSQ master mold to create a thin film. The coated master mold was heated on a hot plate at 180 °C for 10 min and then reverse imprinted on a glass substrate with the help of an adhesive polymer to produce the nanoimprinted photonic structures. The nanoimprinted high-refractive index nanostructure was coated with a film of QDs on PMMA deposited by spin coating.

### Fluorescence microscope imaging

Fluorescence microscopy of nanocomposite films was performed using an Olympus X-81 inverted microscope using a xenon lamp (U-LH75XEAPO) for epi-fluorescence illumination. A standard fluorescence filter cubes (Texas RedTM: excitation wavelength 542–582 nm, dichroic cut-on wavelength 593 nm, emission wavelength 604–644 nm) were used, and fluorescence images were recorded by a CCD camera (Orca II, Hamamatsu).

### Simulations of resonant effects in nanocavity

The nanocavity was simulated using commercial 3D FDTD code (Lumerical). A perfectly matching layer was created on each boundary. The distance between each boundary and the cavity structure is 1 µm. The mesh is uniform, and the mesh size is 20 × 20 × 20 nm. To calculate the photoluminescence decay rate, a dipole source was put in the cavity center. The dipole is oriented along the photonic crystal grooves (TE polarization). The dipole is enclosed by 6 transmission monitors to measure the output power of the dipole.

### Confocal microscope measurements

A confocal microscope system was constructed using a Nikon Eclipse Ti-U microscope with a Nikon 40x objective and a three axis MadCityLabs NanoPDQ piezo nanopositioning sample stage. Supercontinuum (Leukos) passed through the acoustooptic modulator to select a 520 nm wavelength used as the excitation source. The repetition rate is 20 MHz; the average power is 5 mW. Photoluminescence was collected through a 100 μm pinhole into detection optics. For spectral collection we used an Acton 2300i spectrometer.

## Electronic supplementary material

Supplemental Information
