# Supplementary material for: Nanoimprinted High-Refractive Index Active Photonic Nanostructures Based on Quantum Dots for Visible Light
Source: Sci Rep. 2017 Dec 15;7:17645. doi: 10.1038/s41598-017-17732-0 (PMC5732276; doi:10.1038/s41598-017-17732-0)
Supplement: Supplementary file 1 — Supplemental Information [file 41598_2017_17732_MOESM1_ESM.pdf]

# Supporting Information

## Nanoimprinted High-Refractive Index Active Photonic Nanostructures Based on Quantum Dots for Visible Light

**Carlos Pina-Hernandez,[1] Alexander Koshelev,[1] Scott Dhuey,[2] Simone Sassolini,[2] Michela Sainato,[2] Stefano Cabrini,[2] Keiko Munechika[1]**

[1]aBeam Technologies, 22290 Foothill Blvd, St. 2 Hayward, CA, 9454, USA. [2]The Molecular Foundry, Lawrence Berkeley National Laboratory, 1 Cyclotron Road, Berkeley, CA 94720, USA. Correspondence and requests for materials should be addressed to K.M. (email: km@abeamtech.com)

Supporting Information 1:

The photoluminescence of synthesized CdSe–CdS quantum dots was measured before (blue) and after annealing at 180°C (red). The measured data showed photoluminescence spectra centered at around 630 nm with negligible changes in both intensities and line widths, indicating that the quantum dots are optically stable after annealing at 180°C. The PL data before and after annealing was taken from the same QD film.

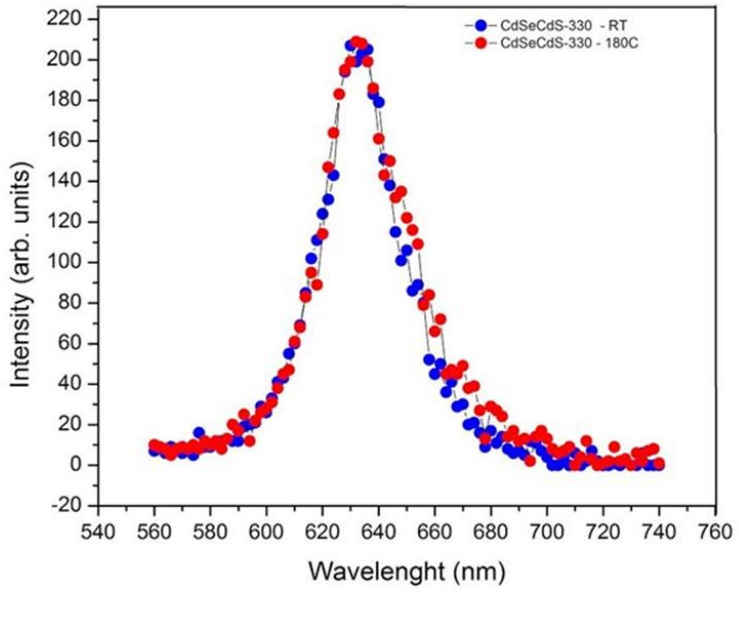


SI Figure 1: Measured PL spectra of CdSe/CdS QDs before and after annealing at 180°C
